# The effects of Kinesio Taping on the trajectory of the forelimb and the muscle activity of the *Musculus brachiocephalicus* and the *Musculus extensor carpi radialis* in horses

**DOI:** 10.1371/journal.pone.0186371

**Published:** 2017-11-22

**Authors:** Antonia Zellner, Barbara Bockstahler, Christian Peham

**Affiliations:** Movement Science Group, Department for Companion Animals and Horses, University of Veterinary Medicine, Vienna, Austria; Massewy University, NEW ZEALAND

## Abstract

**Background information:**

The present study aimed to investigate the effects of Kinesio Taping on the trajectory of the forelimb and the muscle activity of the *M*. *brachiocephalicus* and the *M*. *extensor carpi radialis* in horses.

19 horses and ponies of different breeds (body weight: 496±117 kg), gender (8 mares, 10 geldings and 3 stallions) and ages (14.9±6.9 years old) were analysed without Kinesio Tape (“no tape”), with Kinesio Tape (muscle facilitation application on both muscles of both sides, “with tape”) and immediately after Kinesio Taping (“post tape”) through kinematic motion analysis and surface electromyography on a treadmill at the walk (speed: 1.5±0.1 m/s) and trot (speed: 3.1±0.3 m/s).

**Results:**

The results of the surface electromyography (maximum muscle activity at the walk and trot) and the kinematic motion analysis (maximum stride length and maximum height of the forelimbs flight arc at the walk and trot) showed that there were no significant differences between "no tape", "with tape" and "post tape".

**Conclusion:**

To sum up, Kinesio Taping on the *M*. *brachiocephalicus* and the *M*. *extensor carpi radialis* does not affect (in a positive or negative manner) the trajectory of the forelimb or the muscle activity of the *M*. *brachiocephalicus* and the *M*. *extensor carpi radialis* in horses.

## Introduction

Dr. Kenzo Kase’s Kinesio Taping Technique has been used on humans as a preventative measure in competitive sports and in rehabilitation for many years now. More recently, there has been a lot of hype surrounding the discovery of Equine Kinesio Taping’s potential use on horses.

The Kinesio Taping Method is rumoured to relieve pain through neurological suppression, to create more space under the skin in order to improve blood and lymph flow by eliminating extra fluid, edema or bleeding beneath the skin, to increase proprioception by providing constant cutaneous afferent stimulation through the skin, to realign fascial tissue function by normalising muscle tension and relieving muscle spasms [1].

The question whether or not certain Kinesio Taping Application Techniques might support these claims has, in recent years, been a subject of controversial debate. Several studies on humans agree with the Kinesio Tape’s expected benefits, such as improved muscle strength [2, 3, 4, 5, 6], improved muscle activity [5, 7], increased range of motion [5, 8, 9], scar healing [10], increased lymph flow in rabbits [11] and reduced pain [5, 8, 9, 12].

However, other studies argue that the effects of the Kinesio Tapes are too minor to be clinically relevant [8, 13, 14] or show no changes in muscle strength [15, 16, 17, 18, 19, 20], muscle activity [15, 19, 21, 22], range of motion [14, 19], reduced pain [14] and proprioception [14, 19, 23].

Nevertheless, no studies have measured the effectiveness of Kinesio Taping on horses or on animals in general, except Shim et al. [11], who examined the use of the Kinesio Tape in conjunction with and without passive exercise to promote lymphatic flow in the rabbit’s hind leg by effective and periodic skin deformation. The increase of lymph flow rate due to Kinesio Taping was significant only in conjunction with passive exercise (P = 0.0317). The lymph flow rate increased linearly as the area of Kinesio Taping was increased (P = 0.0011) and the lymph flow rates were significantly different according to site (P = 0.0017).

Ramon et al. [24] investigated the effects of athletic taping (not Kinesio Taping!) of the fore fetlock joint on distal limb kinematics and ground reaction forces of six horses.

Three different adhesive tapes were placed one above the other to stabilise, to maintain or to strengthen the distal limbs`soft tissue structures. A statistically significant interaction was identified for the fetlock during the swing phase (P<0.05) compared with no differences across conditions for the other joints. Peak vertical force reduced significantly (P<0.05) with athletic taping. Athletic taping of the fetlock does not alter the kinematics of the forelimb during stance, but does limit flexion of the fetlock during the swing phase. The decreased peak vertical force may be due to an increased proprioceptive effect.

Dr. Kase suggests that the Kinesio Tape can be used to modulate muscle tone if the Kinesio Tape is applied from muscle origin to muscle insertion [1].

The precise physiological mechanisms underlying the proposed greater motoneuron recruitment have not yet been elucidated and the physiological explanation behind Kinesio Taping application is scarce. However, it is an exciting prospect to be able to positively affect a muscle’s function via an intervention as simple as the external application of a tape.

Therefore, the present study aimed to investigate the effects of Kinesio Taping on the trajectory of the forelimb and the muscle activity of the *M*. *brachiocephalicus* and the *M*. *extensor carpi radialis* in horses.

Consequently, the purpose and hypothesis of this study was that the Kinesio Tape causes an increase in muscle activity of the *M*. *brachiocephalicus* and the *M*. *extensor carpi radialis* on the one hand, and an increase in stride length and in the height of the arc of hoof flight on the other hand.

## Materials and methods

### Horses

This animal experiment was approved by the Ethics Committee of the University of Veterinary Medicine, Vienna (Reference: 68.2505/0160-II/36/2012) and the permissions from the owners to use the horses and ponies exist (S1 Appendix).

A power analysis was performed to determine the sample size. The power analysis was conducted in G-POWER using an alpha of 0.05, a power of 0.80 and an effect size of 0.4. Based on the aforementioned assumptions, the required sample size was determined to be 15.

Therefore, 19 horses and ponies of different breeds (body mass: 496±117 kg), gender (8 mares, 9 geldings and 2 stallions) and ages (14.9±6.9 years old) were used in this study.

The horses were clinically free of lameness. 8 of them were show horses (Pony Mounted Games and eventing horses), also used for training of riders on basic to advanced levels in a riding school, and 11 of them were from the teaching herd of the University for Veterinary Medicine Vienna.

All of them were accustomed to the experimental set-up on the treadmill (Mustang 2200)^1^.

The horses were accommodated in an own stall in the stable of the University of Veterinary Medicine Vienna for one to two days while their measurements were taken. During their stay they got hay and water ad libitum and their common concentrated feedingstuffs.

This animal experiment was approved by the Ethics Committee of the University of Veterinary Medicine, Vienna (Reference: 68.2505/0160-II/36/2012).

### Data collection

**Fig 1:**
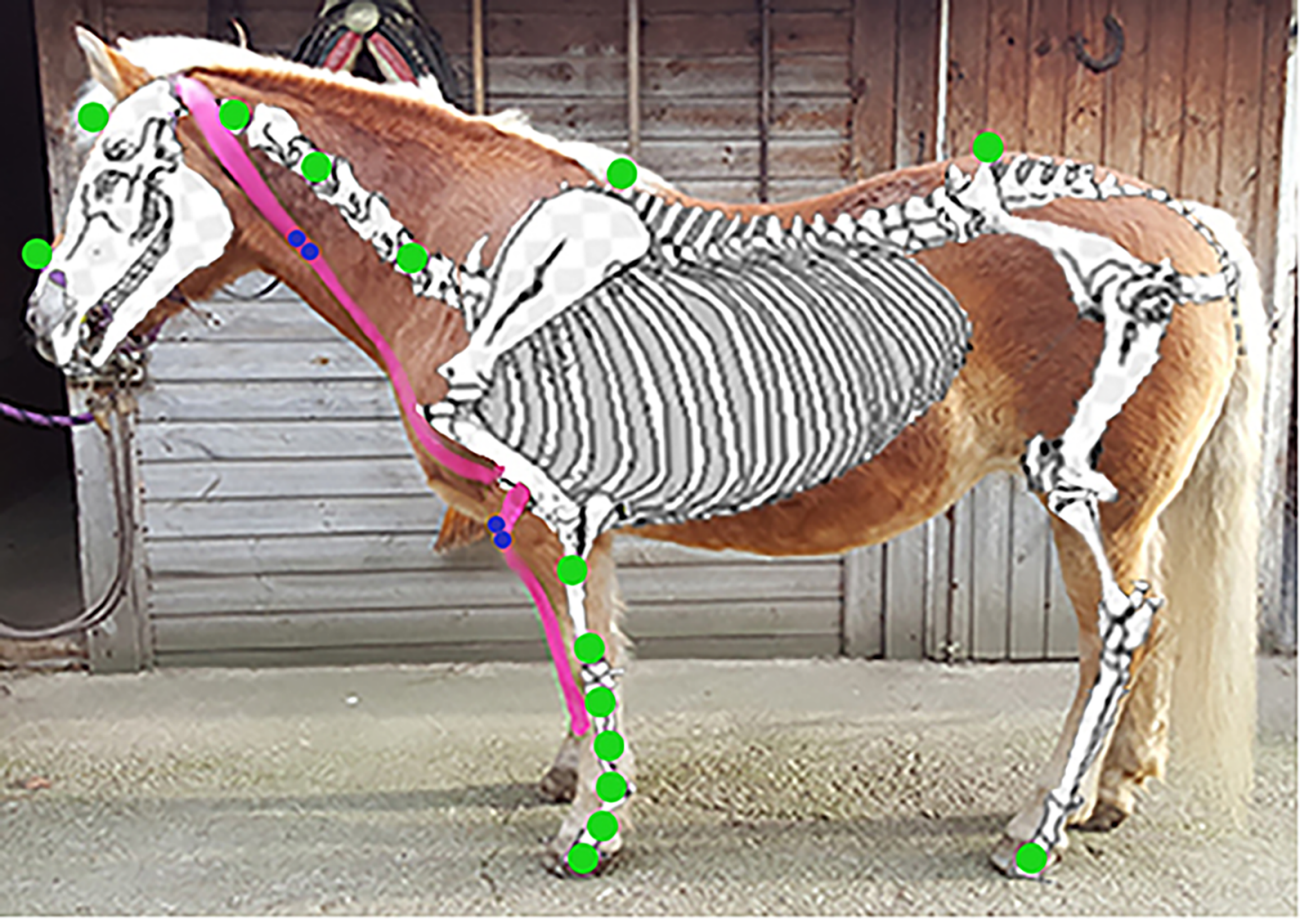
The complete marker set (green dots), the bipolar AgCl electrodes (blue dots) and the Kinesio Tapes (red stripes) on the left side of the horse’s body (lateral view).

26 passive retroreflective skin markers (Fig 1) were placed on anatomical landmarks and at specific levels on the dorsal midline on each horse using adhesive tape.

The 3-dimensional kinematic data was collected by using ten infrared cameras (Eagle Digital RealTime System)^2^ recording at 120 Hz.

For the surface electromyography (sEMG) measurements, the skin (a rectangle measuring 8 x 4cm) over the M. brachiocephalicus and the M. extensor carpi radialis was shaved and two self-adhesive, pre-gelled bipolar AgCl surface electrodes (30 mm in diameter) were fixed bilaterally and parallel to the direction of the muscle fibres over each muscle (Fig 1).

The measurements were taken and the data was transmitted by a telemetric system (Telemyo Mini 16)^3^ (sample frequency 1.2 kHz) to a computer.

The EMG data was synchronised with kinematic software (Cortex 1.4)^2^ to obtain simultaneous recordings.

In order to analyse the gaits without stress, some pre-experimental exercise sessions are required to accustom the horses to this unusual exercise condition [25].

So the horses were trained to walk and trot on the treadmill for a minimum of three times before the measurement started. Within a training session, a minimum of 5 minutes of walking and a minimum of 5 minutes of trotting was required to reach a steady state of locomotion. Between the training sessions the horses had a break in their stalls.

After these at least three pre-experimental exercise sessions the measurements started.

The speed of the treadmill was set between 1.2 m/s (4.3 km/h) and 1.7 m/s (6.0 km/h) for walk and between 1.8 m/s (6.4 km/h) and 3.7 m/s (13.3 km/h) for trot, so each horse walked (1.5±0.1 m/s) and trotted (3.1±0.3 m/s) at its own optimum speed [26]. A standard speed for all horses could not be used because the subjects had a different height at the wither, frame size, body mass, exterior, phlegma and different training conditions. Therefore, the speed was selected for and adapted to each horse, so that the respective horse could show a good working walk and working trot. The individual chosen optimum speed was then used for all measurements (“no tape”, “with tape”, “post tape”).

The advantage of the treadmill was that the horses could be moved for several times at a constant speed on a straight line to achieve optimum measurement results.

The data collection comprised three trials (each 10 sec. including a minimum of 12 motion cycles) at each gait (first walk then immediately trot) in the three different cases without Kinesio Tape (“no tape”), with Kinesio Tape (“with tape”) and without Kinesio Tape again (“post tape”). The recording started at each gait immediately after reaching the steady state of locomotion. Even the experienced and trained horses took at least 1 minute for their gait pattern to stabilize each time after a change of speed.

Immediately after the measurement "no tape" (three times 10 seconds walk, three times 10 seconds trot), the treadmill stopped and the two muscles *M*. *brachiocephalicus* and *M*. *extensor carpi radialis* were taped with an I-shaped Kinesio Tape strip from muscle origin to muscle insertion (muscle facilitation application on both muscles of both sides) on the surface of the skin. The “with tape” measurement (again three trials–each 10 seconds for walk and for trot) was taken 30 minutes after the Kinesio Taping application, because proponents of the Kinesio Taping technique claim that 30 minutes is the time it takes for the definite onset of the reflex loop. During this time we kept the horses waiting in their stalls.

Immediately after the measurement “with tape”, the Kinesio Tapes were removed and the “post tape” measurement started (again three trials–each 10 seconds for walk and for trot).

Always the same handler was placed in front of the horse on the horse`s left side during the training and measurements on the treadmill. The horses wore halters with a loose rope for holding their heads free in their natural positions.

### Kinesio Taping application

The horses’ skin was clean and free of perfumes, oils and lotions.

The Kinesio® Tex Gold™FP^4^ is a latex-free hypoallergenic cotton fibre tape with an acrylic heat-activated backing that only stretches along its longitudinal axis.

The Kinesio Tape ends were rounded to prevent the square edges from peeling off.

Immediately after the measurement "no tape", the two muscles M. brachiocephalicus and M. extensor carpi radialis were taped with an I-shaped Kinesio Tape strip from muscle origin to muscle insertion (muscle facilitation application on both muscles of both sides).

The Facilitation Application Taping Method is used to optimise the muscle function [1].

### Data analysis and processing of the kinematic data

The 3-dimensional coordinates of each marker during the time course of each experiment were calculated from 2-D videos of the cameras using kinematic (Cortex 1.4)^2^ software. These time series were then smoothed by using a Butterworth low-pass filter (cut-off frequency, 10 Hz). The data was split into motion cycles by MATLAB^5^ using the left forelimb as reference. The duration of each motion cycle was calculated. The sEMG signal was rectified and low-pass filtered by reduction of the sample rate to 120 Hz so that motion and sEMG were comparable (same timescale). Then the EMG signal was filtered using a Butterworth low-pass filter (cut-off frequency, 10 Hz). The same procedure was used for the kinematic time curves.

The mean and standard deviation of the maximum muscle activity, the maximum stride length and the maximum height of the forelimbs flight arc were calculated from all the measured motion cycles for each horse. The maxima were calculated as local maxima within the first and second half of the motion cycle. The occurrence of the maxima within the motion cycle is presented in percent of the duration of the motion cycle.

The stride length (SL = path A + path B) of a motion cycle on the treadmill was defined as the forward distance (= path A) between the ground contact of the forehoof and the next similar position of the same forehoof in gait (walk or trot) plus the backward distance (= path B) of the ipsilateral forehoof during stance phase on the treadmill (the forehoof moved back during stance phase).

The marker on the lateral hoof wall of the left forehoof was used for the stride length of the left forehand and the marker on the lateral hoof wall of the right forehoof was used for the stride length of the right forehand.

The height of the forelimbs flight arc of a motion cycle on the treadmill (from lift-off to the hoof landing) is defined as the maximum distance (height) of the forehoof (the marker on the lateral hoof wall of the forehoof) to the ground.

The marker on the lateral hoof wall of the left forehoof was used for the maximum height of the left forelimbs flight arc and the marker on the lateral hoof wall of the right forehoof was used for the right forelimbs flight arc.

### Statistical analysis

The statistical analyses were performed using SPSS 20.0^6^. The normal distribution of data was tested by the use of a Kolmogorov-Smirnov test.

Differences between left and right sides were tested using a Student`s t*-*test for paired samples.

Associations between “no tape”, “with tape” and “post tape”, walk and trot, were evaluated by ANOVA for repeated measures with a Bonferroni post hoc test.

Values of P<0.05 were considered significant.

## Results

The mean values of the maximum muscle activity, stride length and height of the forelimbs flight arc, which were measured in this study, are presented in Table 1, Table 2 and Table 3.

**Table 1:** Results of the sEMG of the M. brachiocephalicus—the mean values and S.D. are listed.

			M. brachiocephalicus (EMG activity)				
			right			left	
		no tape	with tape	post tape	no tape	with tape	post tape
walk	Maximum1 (mv)	18.64±2.68	20.52±6.05	17.37±3.36	7.38±0.96	6.66±1.03	7.00±0.63
	Maximum2 (mv)	8.78±1.58	6.43±0.80	6.19±0.54	18.11±2.58	16.62±2.21	16.04±2.04
trot	Maximum1 (mv)	31.42±5.15	30.55±5.94	33.69±6.90	28.16±3.30	30.52±3.70	30.73±3.88
	Maximum2 (mv)	30.89±3.78	33.08±5.30	36.17±5.98	23.58±2.66	23.92±2.42	24.75±2.69

**Table 2:** Results of the sEMG of the M. extensor carpi radialis—the mean values and S.D. are listed.

		M. extensor carpi radialis (EMG activity)		
		no tape	with tape	post tape
walk	Maximum (mv)	29.71±1.89	27.10±1.67	25.43±1.79
trot	Maximum (mv)	61.80±3.94	58.18±2.90	59.21±3.21

**Table 3:** Results of the kinematic motion analysis—the mean values and S.D. are listed.

			Stride length	
		no tape	with tape	post tape
walk	Maximum (m)	1.55±0.03	1.56±0.04	1.54±0.05
trot	Maximum (m)	2.06±0.06	2.04±0.06	2.02±0.06
			The height of the arc of hoof flight	
		no tape	with tape	post tape
walk	Maximum (mm)	82.85±6.79	79.75±7.08	79.53±7.13
trot	Maximum (mm)	111.34±10.24	106.71±9.66	105.74±10.02

### M. brachiocephalicus

**Fig 2:**
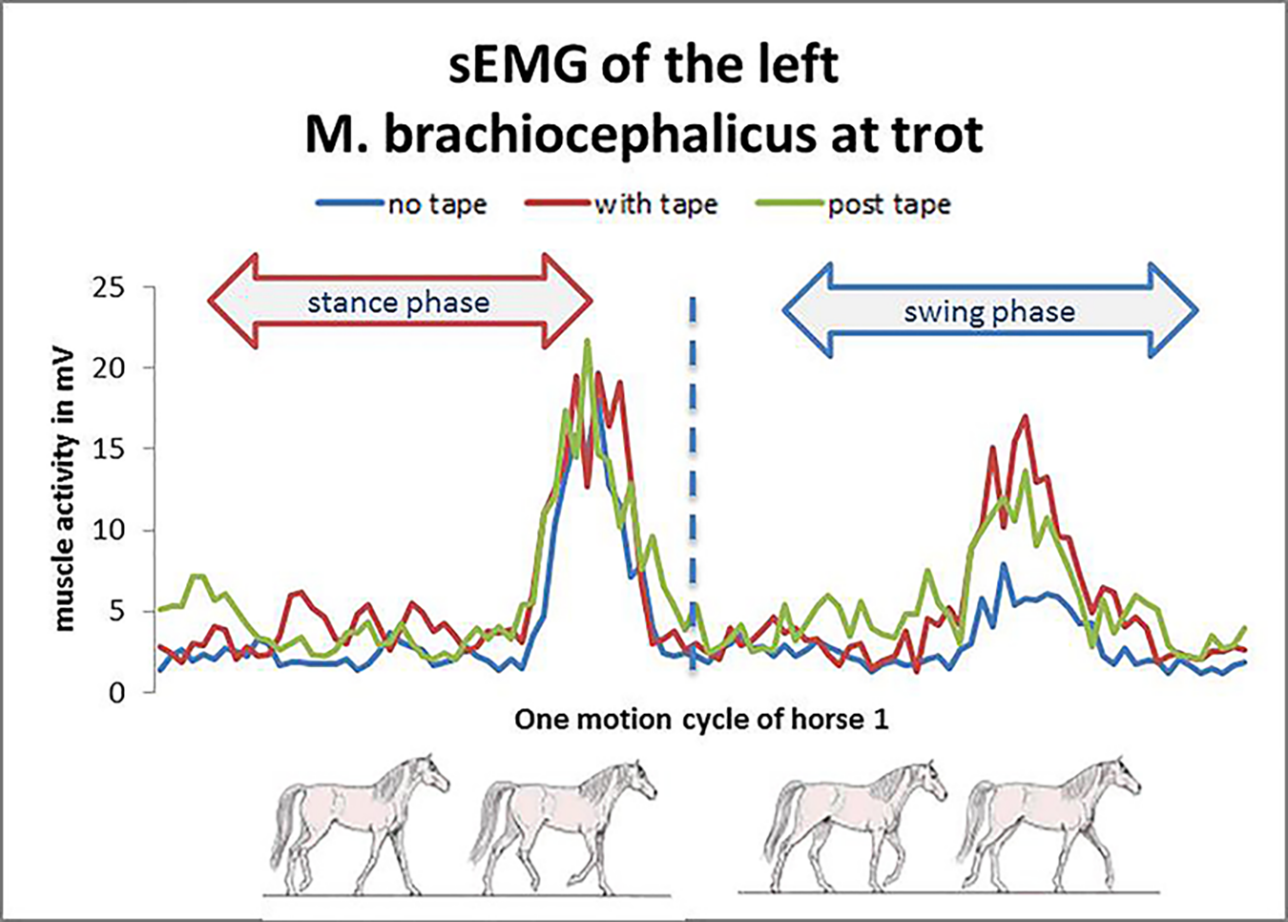
Example for a sEMG of the M. brachiocephalicus. The M. brachiocephalicus muscle activity was highest during the later part of the ipsilateral forelimb stance phase.

The phasic pattern of the *M*. *brachiocephalicus* showed two bursts of EMG activity per stride (Fig 2).

The *brachiocephalicus* muscle activity was high during the later part of the ipsilateral forelimb stance phase and during the mid-late swing phase (Fig 2).

The results of the surface electromyography (maximum muscle activity at the walk and trot) of the *M*. *brachiocephalicus* showed that there were no significant differences between "no tape", "with tape" and "post tape" (P>0.05).

However, the gait had a significant impact on the amount of muscle activity. At the trot, the muscle activity of the *M*. *brachiocephalicus* increased significantly (P = 0.00).

Even though this study was not set up to test the laterality in horses, additional significant differences between the first muscle activity maximum of the left and the right *M*. *brachiocephalicus* at walk (P = 0.03) and the second muscle activity maximum at walk (P = 0.01) and at trot (P = 0.02) were discovered.

### M. extensor carpi radialis

**Fig 3:**
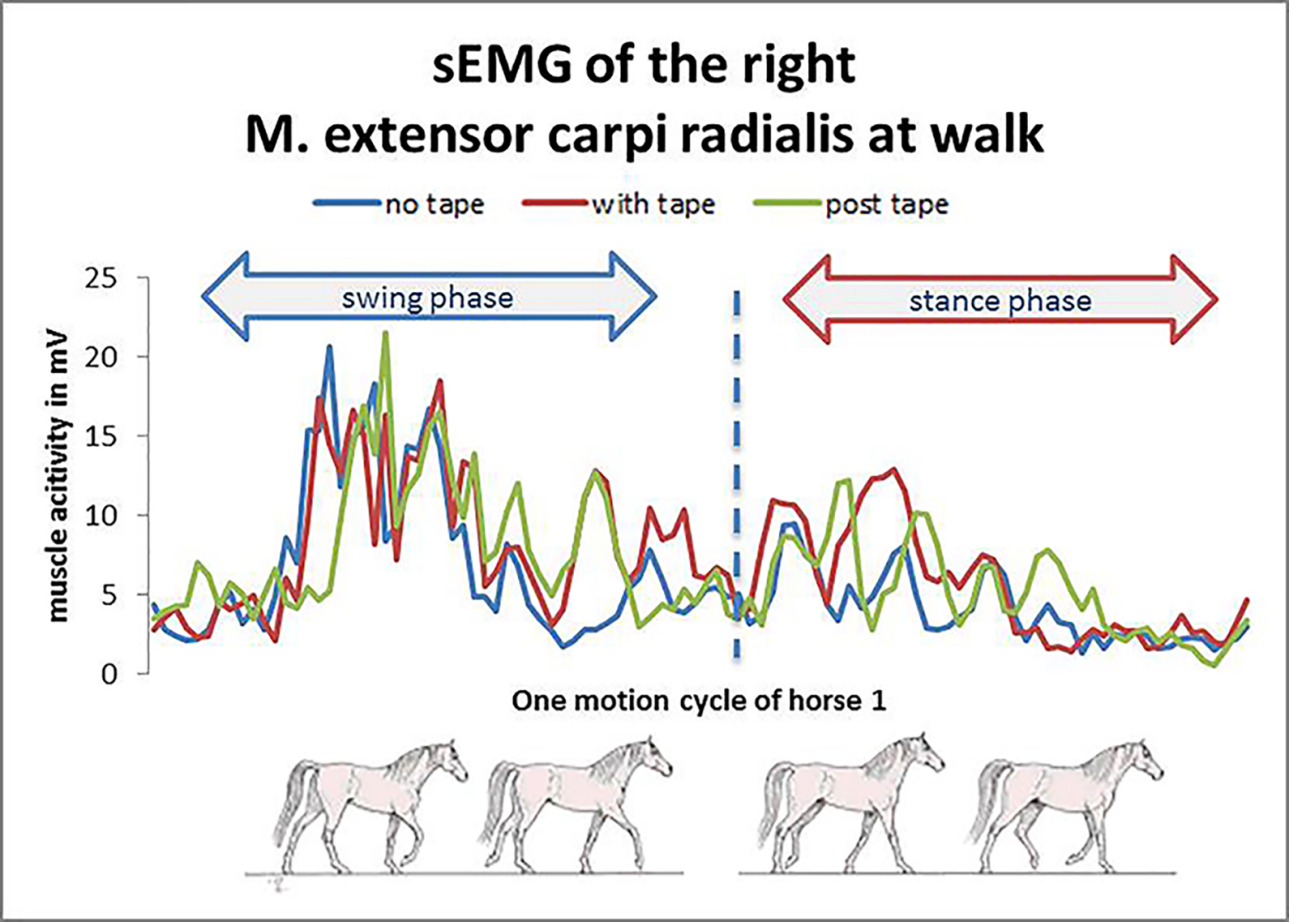
Example for a sEMG of the M. extensor carpi radialis. The electromyographic activity in extensor carpi radialis was concentrated at the middle of the swing phase.

The *M*. *extensor carpi radialis* showed a unique burst of EMG activity per stride (Fig 3). The electromyographic activity in the *M*. *extensor carpi radialis* was concentrated at the middle of the swing phase.

The results of the surface electromyography (maximum muscle activity in mV at the walk and trot) of the *M*. extensor carpi radialis showed that there were no significant differences between "no tape", "with tape" and "post tape" (P>0.05).

However, the gait had a significant impact on the amount of muscle activity. At the trot, the muscle activity of the *M*. *extensor carpi radialis* increased significantly (P = 0.00).

### The stride length

**Fig 4:**
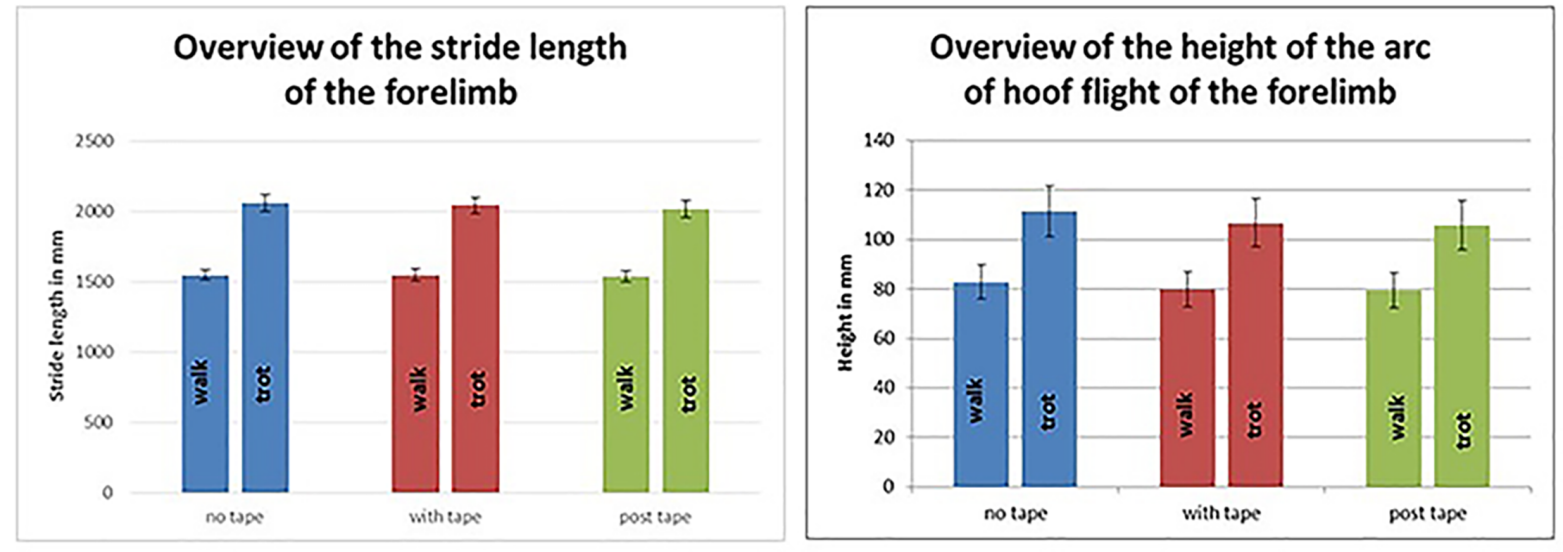
The results of the kinematic motion analysis (maximum stride length in m and maximum height of the arc of hoof flight in mm at the walk and trot) of the forelimbs showed that there were no significant differences between "no tape", "with tape" and "post tape" (P>0.05).

The results of the kinematic motion analysis (maximum stride length in mm at the walk and trot) of the forelimbs showed that there were no significant differences between "no tape", "with tape" and "post tape" (P>0.05) (Fig 4).

However, the gait had a significant impact on the stride length. At the trot, the stride length increased significantly (P = 0.00).

### The height of the arc of hoof flight

The results of the kinematic motion analysis (maximum height of the arc of hoof flight at the walk and trot) of the forelimbs showed no significant differences between "no tape", "with tape" and "post tape" (P>0.05) (Fig 4).

However, the gait had a significant impact on the step height. At the trot, the height of the arc of hoof flight increased significantly (P = 0.00).

## Discussion

The expectation and the hypotheses of the present study were that the Kinesio Tape would cause an increase in muscle activity on the one hand, and an increase in stride length and in the height of the arc of hoof flight on the other hand, because Dr. Kenzo Kase describes increasing proprioception by providing constant cutaneous afferent stimulation through the skin [1]. Proponents of the technique speculate that the cutaneous afferent stimulation of the slowly adapting type 2 mechanoreceptors located deep in the dermis, provided by the Kinesio Tape, might induce greater motor unit recruitment, which results in an increase in muscle strength [23].

These hypotheses could not be confirmed, because the results of the surface electromyography (maximum muscle activity at the walk and trot) and the kinematic motion analysis (maximum stride length and maximum height of the arc of hoof flight of the forelimbs at the walk and trot) showed that there were no significant differences between "no tape", "with tape" and "post tape" (P>0.05).

Our results are in line with previous studies in humans that did not find any significant change in muscle activity [15, 19, 21, 22] after Kinesio Taping application. For example, Cai et al. [15] found no significant differences in the electromyographic activity and self-perceived performance between different application techniques of Kinesio Taping (facilitatory Kinesio Taping, inhibitory Kinesio Taping, no Kinesio Taping) in 31 healthy participants. Grip strength was quantified by a dynamometer and the electromyographic activity of the wrist extensors were measured using sEMG.

In contrast, some studies have indicated a positive effect of Kinesio Taping application on muscle strength and muscle activity. In the study of Slupik et al. [7], the electromyographic recruitment of the vastus medialis increased significantly after Kinesio Taping application. The results presented by Fratocchi et al. [4] suggest that Kinesio Taping application, applied over the biceps brachii, increases concentric elbow peak torque in a population of 20 healthy participants, if compared with a Placebo Tape.

Furthermore, this study demonstrated that the gaits (walk and trot) have a significant impact on the amount of muscle activity (P = 0.00), the stride length (P = 0.00) and the height of the arc of hoof flight (P = 0.00). At the trot, the muscle activity of the *M*. *brachiocephalicus* (P = 0.00) and the *M*. *extensor carpi radialis* (P = 0.00), the stride length (P = 0.00) and the height of the arc of hoof flight (P = 0.00) increased significantly. This conforms with previous reports: an increase in speed led to an increase in muscle activity of the *M*. *brachiocephalicus* [27, 28], the long head and the lateral head of the *M*. *triceps* [27, 29], the *M*. *gluteus medius*, the *M*. *tensor fascia lata and the M*. *longissimus dorsi* [29], the M. splenius [29, 30], the *M*. *rectus abdominis* [29, 31] and the *M*. *obliquus externus abdominis* [31].

In the present study, the EMG of the *M*. *brachiocephalicus* was high during the later part of the ipsilateral forelimb stance phase and during the suspension phase in order to achieve protraction of the ipsilateral forelimb. The second burst was in the middle of the swing phase during locomotion (Fig 2).

Tokuriki and Aoki [32] also reported that the M. brachiocephalicus is a limb and a neck muscle. Their measured EMG activity occurred from mid to late stance phase to mid to late swing phase during locomotion with and without a rider. Such a long period of activity indicates that the *M*. *brachiocephalicus* probably fulfils a complex role during locomotion. Tokuriki et al. [33] described a higher EMG of the *M*. *brachiocephalicus* while swimming, which indicates that the muscle is significantly involved in the anteversion of the forelimb against water resistance. Hodson-Tole [27] also reported that the *M*. *brachiocephalicus* was active in the terminal part of the stance phase and is therefore also definitely responsible for the protraction of the forelimbs.

Even though this study was not set up to test the laterality in horses, additional significant differences between the first muscle activity maximum of the left and the right *M*. *brachiocephalicus* at walk (P = 0.03) and the second muscle activity maximum at walk (P = 0.01) and at trot (P = 0.02) were discovered. Horses are commonly led from the left side–as in this study during exercise on the treadmill, which possibly generated the laterality. Another possibility for the laterality of horses is the preferential use of muscles on one side as a consequence of handedness [34].

In future studies, positioning two handlers, one on the left side and one on the right side, or changing the position in case of one handler, may be helpful in order to reduce this influence.

In contrast to the *M*. *brachiocephalicus*, the muscle activity of the M. *extensor carpi radialis* has not been well investigated yet. In the present study, the sEMG of the *M*. *extensor carpi radialis* is concentrated at the middle of the swing phase for controlling carpal extension to protract the limb (Fig 3). The onset of the limb extension, which readies the limb for ground contact during the late swing phase, is mainly performed by the extensor muscles of the antebrachium.

Measurements with sEMG and kinematic motion analysis enable a very accurate representation of muscle function and motion patterns.

The disadvantage of these methods is that the measurements are only feasible in motion analysis laboratories using special equipment.

Another disadvantage could be the use of the treadmill. Each horse was urged to move at the same speed during “no tape”, “with tape” and “post tape” measurement. So it cannot be ruled out that the horses would have chosen a faster speed “with tape”.

The “with tape” measurement was taken 30 minutes after Kinesio Taping application. It is possible that repeated applications of Kinesio Taping, or a longer period of application, may be necessary to detect changes in muscle strength. In other studies, some Kinesio Taping effects were observed after Kinesio Taping application for as long as one or two days [7, 9]. Slupik et al. [7] determined in 27 healthy persons the effect of Kinesio Taping on changes in the tone of the vastus medialis muscle during isometric contractions. An examination performed 24 hours after the placement of the Kinesio Tape revealed significantly increased recruitment of the muscle's motor units, as expressed by peak torque (p = 0.0005). An examination after 72 hours of Kinesio Taping revealed a statistically significant increase in bioelectrical activity of the muscle compared to baseline (p = 0.0015). A decrease in peak torque was observed in 6 participants. A measurement taken after 96 hours of Kinesio Taping did not show a significant change in peak torque (p = 0.93).

In contrast, Thelen et al. [9] determined the short-term clinical efficacy of Kinesio Tape (KT) when applied to college students with shoulder pain, as compared to a sham tape application. 42 subjects clinically diagnosed with rotator cuff tendonitis/impingement were randomly assigned to 1 of 2 groups: therapeutic KT group or sham KT group. Subjects wore the tape for 2 consecutive 3-day intervals. Self-reported pain and disability and pain-free active range of motion (ROM) were measured at multiple intervals to assess for differences between groups. The therapeutic KT group showed immediate improvement in pain-free shoulder abduction (P = 0.005) after tape application. No other differences between groups regarding ROM, pain, or disability scores at any time interval were found.

This time issue warrants further investigation.

Furthermore, the difference between the naked skin of humans and the coat of horses should be mentioned, although Dr. Kenzo argues that the coat does not affect the effects of Kinesio Taping.

A limiting factor of this study is the relatively small number of horses. To acquire a larger amount of data, it would have been desirable to measure a greater number of subjects. Participation in such a study requires great cooperation by the horse owners. They have to bring their horses to where the measurements are being performed for at least one day. It is also very time consuming to get the horses accustomed to the new situation on the treadmill.

Another limiting factor is that there was no separate control group in the present study and the horses varied in size and conformation. Future research requires an adequate and generalised sample size, sample population (same breed and purpose of use) and the incorporation of a true control group.

## Conclusion

To sum up, Kinesio Taping on the *M*. *brachiocephalicus* and the *M*. *extensor carpi radialis* does not affect (in a positive or negative manner) the trajectory of the forelimb and the muscle activity of the *M*. *brachiocephalicus* and the *M*. *extensor carpi radialis* in horses.

Additional research on horses is required before any conclusive statement can be made with regard to the recommended use of Kinesio Taping and its effects on muscle strength or athletic performance.

Likewise, further examination of horses is required in order to explore the physiological and therapeutic effectiveness of the Kinesio Taping Technique for relieving pain, improving blood and lymph flow, increasing proprioception, realigning fascial tissue function and relieving muscle spasms.

## Supporting information

S1 Appendix Permission of the horse owners. (PDF)

S1 Dataset (XLSX)

## References

[1] Kase K, Wallis J, Kase T. Clinical therapeutic application of the Kinesio Taping Method. 3rd edition Albuquerque, NM: Kinesio IP, LLC; 2013.

[2] Aktas G, Baltaci G. Does kinesiotaping increase muscles strength and functional performance? Isokinetics and Exercise Science 2011;19:149–155.

[3] Donec V, Varzaityte L, Krisciunas A. The effect of Kinesio taping on maximal gripforce and key pinch force. Polish Annals of Medicine 2013;19:98–105

[4] Fratocchi G, Di Mattia F, Rossi R, Mangone M, Santilli V, Paoloni M. Influence of Kinesio taping applied over biceps brachii on isokinetic elbow peak torque. A placebo controlled study in a population of young healthy subjects. Journal of Science and Medicine in Sport 2013;16:245–249. doi: 10.1016/j.jsams.2012.06.003 22771110 10.1016/j.jsams.2012.06.003

[5] Hsu YH, Chen WY, Lin HC, Wang WT, Shih YF. The effects of taping on scapular kinematics and muscle performance in baseball players with shoulder impingement syndrome. Journal of Electromyographie and Kinesiology 2009;19:1092–1099. 10.1016/j.jelekin.2008.11.003 19147374

[6] Vithoulka I, Beneka A, Malliou P, Aggelousis N, Karatsolis K, Diamantopoulos K. The effects of Kinesio Taping on quadriceps strength during isokinetic exercise in healthy non athlete women. Isokinetics and Exercise Science 2010;18:1–6.

[7] Slupik A, Dwornik M, Bialoszewski D, Zych E. Effect of KinesioTaping on bioelectrical activity of vastus medialis muscle. Preliminary report. 2007;9:644–651. 18227756

[8] Gonzalez-Iglesias J, Fernandez-de-Las-Penas C, Cleland JA, Huijbregts P, Del Rosario Gutierrez-Vega M. Short-term effects of cervical Kinesio taping on pain and cervical range of motion in patients with acute whiplash injury: a randomized cinical trial. Journal of Orthopaedic & Sports Physical Therapy 2009;39:515–521. 19574662 10.2519/jospt.2009.3072

[9] Thelen MD, Dauber JA, Stoneman PD. The clinical efficacy of Kinesio Tape for shoulder pain: a randomized, double-blinded, clinical trial. Journal of Orthopaedic & Sports Physical Therapy 2008;38:389–395. 18591761 10.2519/jospt.2008.2791

[10] Karwacinska J, Kiebzak W, Finda-Stepanek B, Kowalski IM, Protasiewicz-Faldowska H, Trybulski R, Starcynska M. Effectiveness of Kinesio Taping on hypertrophic scars, keloids and scar contractures. Polish Annals of Medicine 2012;19:50–57.

[11] Shim J, Lee HR, Lee DC. The use of elastic adhesive tape to promote lymphatic flow in the rabbit hind leg. Yonsei Medical Journal 2003;44:1045–1052. doi: 10.3349/ymj.2003.44.6.1045 14703615 10.3349/ymj.2003.44.6.1045

[12] Aguilar-Ferrandiz ME, Castro-Sanchez AM, Mataran-Penarrocha GA, Garcia-Muro F, Serge T, Moreno-Lorenzo C. Effects of Kinesio taping on venous symptoms, bioelectrical activity of the gastrocnemius muscle, range of ankle motion, and quality of life in postmenopausal women with chronic venous insufficiency: a randomized controlled trial. Archives of Physical Medicine and Rehabilitation 2013;94:2315–2328. doi: 10.1016/j.apmr.2013.05.016 23769763 10.1016/j.apmr.2013.05.016

[13] Bassett KT, Lingman SA, Ellis RF. The use and treatment efficacy of kinaesthetic taping for musculoskeletal conditions: A systematic review. New Zealand Journal of Physiotherapy 2010;38:56–62.

[14] Williams S, Whatman C, Hume PA, Sheerin K. Kinesio taping in treatment and prevention of sports injuries: a meta-analysis of the evidence for its effectiveness. Sports Medicine 2012;42:153–164. doi: 10.2165/11594960-000000000-00000 22124445 10.2165/11594960-000000000-00000

[15] Cai C, Au IPH, An W, Cheung RTH. Facilitatory and inhibitory effects of Kinesio tape: Fact or fad? Journal of Science and Medicine in Sport 2016;19:109–112. doi: 10.1016/j.jsams.2015.01.010 25687484 10.1016/j.jsams.2015.01.010

[16] Chang HY, Chou KY, Lin JJ. Immediate effect of forearm Kinesio taping on maximal grip strength and force sense in healthy collegiate athletes. Physical Therapy in Sport 2010;11:122–127. doi: 10.1016/j.ptsp.2010.06.007 21055705 10.1016/j.ptsp.2010.06.007

[17] Csapo R, Alegre LM. Effects of Kinesiotaping on skeletal muscle strength–a meta-analysis of current evidence. Journal of Science and Medicine in Sport 2015;18:450–456. doi: 10.1016/j.jsams.2014.06.014 25027771 10.1016/j.jsams.2014.06.014

[18] Fu TC, Wong MK, Pei YC, Wu KP, Chouh SW, Lin YC. Effect of Kinesio Taping on muscle strength in athletes–a pilot study. Journal of Science and Medicine in Sport 2008;11:198–201. doi: 10.1016/j.jsams.2007.02.011 17588814 10.1016/j.jsams.2007.02.011

[19] Gómez-Soriano J, Abián-Vicén J, Aparicio-García C, Ruiz-Lazaro P, Simon-Martinez C, Bravo-Esteban E, Fernandez-Rodriguez JM. The Effect of Kinesio Taping on Muscle Tone in Healthy Subjects. A Randomized Double-Blind, Placebo-Controlled Crossover Trial. Manual Therapy 2014;19:131–136. 24829961 10.1016/j.math.2013.09.002

[20] Vercelli S, Sartorio F, Foti C, Colletto L, Virton D, Ronconi G, Ferriero G. Immediate effects of kinesiotaping on quadriceps muscle strength: a single-blind, placebo-controlled crossover trial. Clinical Journal of Sport Medicine 2012;22:319–326. doi: 10.1097/JSM.0b013e31824c835d 22450591 10.1097/JSM.0b013e31824c835d

[21] Cools AM, Witvrouw EE, Danneels LA, Cambier DC. Does taping influence electromyographic muscle activity in the scapular rotators in healthy shoulders? Manual Therapy 2002;7:154–162. 12372312 10.1054/math.2002.0464

[22] De Hoyo M, Álvarez-Mesa A, Sanudo B, Carrasco L, Dominguez S. Immediate effect of kinesio taping onmuscle response in young elite soccer players. Journal of Sport Rehabilitation 2013;22:53–58. 23006787 10.1123/jsr.22.1.53

[23] Halseth T, McChesney JW, DeBeliso M, Vaughn R, Lien J. The effects of Kinesio Taping on proprioception at the ankle. Journal of Sports Science and Medicine 2004;3:1–7. PMC3896108 24497814

[24] Ramon T, Prades M, Armengou L, Lanovaz JL, Mullineaux DR, Clayton HM. Effects of athletic taping on the fetlock on distal limb mechanics. Equine Veterinary Journal 2004;36 (8):764–768. 15656512 10.2746/0425164044848127

[25] Buchner HHF, Savelberg HHCM, Schamhardt HC, Merkens HW, Barneveld A. Habituation of horses to treadmill locomotion. Equine Veterinary Journal 1994;26:13–15.

[26] Peham C, Licka T, Mayr A, Scheidl M, Girtler D. Speed dependency of motion pattern consistency. Journal of Biomechanics 1998:31:769–772. 9802776 10.1016/s0021-9290(98)00040-2

[27] Hodson-Tole E. Effects of treadmill inclination and speed on forelimb muscle activity and kinematics in the horse. Equine and Comparative Exercise Physiology 2006;3:61–72.

[28] Kienapfel K. The effect of three different head-neck positions on the average EMG activity of three important neck muscles in the horse. Journal of Animal Physiology and Animal Nutrition 2014;99:132–138. doi: 10.1111/jpn.12210 24954642 10.1111/jpn.12210

[29] Robert C, Valette JP, Pourcelot P, Audigie F, Denoix JM. Effects of trotting speed on muscle activity and kinematics in saddlehorses. Equine Veterinary Journal 2002;34:295–301. 10.1111/j.2042-3306.2002.tb05436.x 12405704

[30] Zsoldos R, Kotschwar AB, Kotschwar A, Groesel M, Licka T, Peham C. Electromyography activity of the equine splenius muscle and neck kinematics during walk and trot on the treadmill. Equine Veterinary Journal 2010a;42:455–461. 10.1111/j.2042-3306.2010.00263.x 21059045

[31] Zsoldos R, Kotschwar A, Kotschwar AB, Prodriguez CP, Peham C, Licka T. Activity of the equine rectus abdominis and oblique external abdominal muscles measured by surface EMG during walk and trot on the treadmill. Equine Veterinary Journal 2010b;42:523–529. 10.1111/j.2042-3306.2010.00230.x 21059055

[32] Tokuriki M, Aoki O. Neck muscles activity in horses during locomotion with and without a rider. Equine Exercise Physiology 1991;3:146–150.

[33] Tokuriki M, Ohtsuki R, Kai M, Hiraga A, Oki H, Miyahara Y, Aoki O. EMG activity of the muscles of the neck and forelimbs during different forms of locomotion. Equine Veterinary Journal 1999;30:231–234. 10.1111/j.2042-3306.1999.tb05224.x 10659258

[34] Whelan PJ. Electromyogram recordings from freely moving animals. Methods 2003;30:127–141. 12725779 10.1016/s1046-2023(03)00074-4

