# Supplementary material for: The effects of Kinesio Taping on the trajectory of the forelimb and the muscle activity of the Musculus brachiocephalicus and the Musculus extensor carpi radialis in horses
Source: PLoS One. 2017 Nov 22;12(11):e0186371. doi: 10.1371/journal.pone.0186371 (PMC5699842; doi:10.1371/journal.pone.0186371)
Supplement: S1 Appendix — (PDF) [file pone.0186371.s001.pdf]

Information und Einverständniserklärung des Tierhalters

1) Titel und kurze Beschreibung der Studie:

1.) Auswirkung verschieden langer Ausbinder auf die Aktivität des langen Rückenmuskels.
2.) Auswirkung verschieden langer Ausbinder auf die Aktivität der Halsmuskulatur.
3.) Die Zugkräfte zwei verschiedener Trensen beim ausgebundenen Pferd.
4.)Die Zugkräfte bei unterschiedlich festem Nasenriemen beim ausgebundenen Pferd.

Die Messungen für die Arbeiten laufen parallel jeweils im Schritt und Trab:
erste Messung: freie Kopf-Hals-Haltung
zweite Messung: Pferd ist ausgebunden mit der Nasen-Stirnlinie in der Senkrechten
dritte Messung: das Pferd ist mit verkürzten Ausbinder ausgebunden
vierte Messung: Das Pferd ist in der Senkrechten ausgebunden mit sehr eng verschnalltem Nasenriemen
fünfte Messung: Das Pferd ist in der Senkrechte ausgebunden und trägt ein Stangengebiss (die vorangegangenen Messungen mit doppelt gebrochener Trense)

2) Studienziel und kurze Beschreibung der Eingriffe:

1. Hypothese: Hyperflexion hemmt die Aktivität des langen Rückenmuskels sowie der Halsmuskulatur.
2. Hypothese: Die Zugkräfte verstärken sich bei engem Nasenriemen.
3. Hypothese: Die Zugkräfte unterscheiden sich je nach verwendetem Gebiss.

Vorbereitung:
Training an das Laufband
Rasieren der benötigten Stellen (C2, C5 links und rechts, sowie T16, L3 links und rechts
Aufbringen von Elektroden mit Kontaktgel auf die rasierten und mit Alkohol gereinigten Hautstellen
Aufbringen von reflektierenden Markern mit Klebeband auf das Fell
Durchführung:
EMG - Messungen
Zügelkraftmessungen mittels Kraftsensor in den Ausbindern

3) Kosten: Die/Der TierhalterIn trägt die Transportkosten

4) mögliche Nebenwirkungen: Irritation der Haut nach der Rasur

5) Ich, Univ.-Prof. Dr. **René van den Hoven** .

geboren am 25 3 1952

wohnhaft in Marchegg ArbeitsoOrt Wien /**Mitarbeiter der Vetmeduni Wien**

erkläre mich als TierhalterIn der **Übungspferdes der Pferdeklinik**

Diese Übungspferde sind als Versuchstiere schon gemeldet und von Ministerium zur Verwendung der Tiermedizinische Lehre schon genehmigt von Juli 2012 bis Juli 2014 unter Nummer: 68.2505/0160-II/36/2012.

Nr. des Pferdepasses: **siehe TIS.**

hiermit einverstanden, dass die Übungspferdepferde, für die in diesem Informationsblatt beschriebene Untersuchungen herangezogen werden können. Ich wurde darüber informiert, dass mein Pferd für diese Studie an bestimmten Stellen rasiert wird und für den Versuch ausgebunden am Laufband gehen muss.

Datum und Unterschrift:

21-08-2012

Univ.-Prof. dr. René van den Hoven

Pferdeklinik, Abteilung Interne und Infektionsmedizin

Dept 4.

Bei Rückfragen stehe ich gerne zur Verfügung

A. Univ. Prof. DI. Dr. Christian Peham

Movement Science Group

Veterinärmedizinische Universität Wien

Veterinärplatz 1

Tel.01/250 77-5517

Email: christian.peham@vetmeduni.ac.at
